# Pulp Capping

**Published:** 1880-12

**Authors:** 


### EDITORIAL, ETC.

*Pulp Capping*, and the conservative treatment of this organ has perhaps more advocates to-day than ever, and fewer enthusiasts—that is, its desirability is more and more recognized, although its practicability is, except with a few, less confidently asserted. That many cases are successful few, save those who are utterly prejudiced will deny; that it is nearly uniformly successful few now claim. It is true it is well to try what can be done in many cases of exposure, but after all one comes to realize sadly that in cases not a few the safest method and that least liable to give trouble in the end is devitalization and removal.

To those who feel that they wish light on this subject, the method proposed and practiced by Prof. R. B. Winder, of the Baltimore College of Dental Surgery, seems reasonable, and Prof. W. claims is fairly successful. The ordinary steps being taken of careful removal of decay, perfect drying of cavity, etc., a small shred of very fine surgeon's sponge is saturated with liquor plumbi sub. acit., and placed over the point of exposure. Oxychloride of zinc is placed over this, *with no pain resulting*, due probably partly to the anodyne effects of the lead water and partly to the fact that the chloride of zinc is not diluted at the expense of the fluids of the pulp. The soothing and healing properties of the lead are well known, and the fluid is abstracted from the sponge very slowly. It is thought also that the sponge as it gives up its fluid contents retracts and gives slight room for the expansion of the tissues in case of inflammation. Prof. Winder claims that by this method his success is reasonably fair, and he hopes the suggestion may be useful to the profession. H.

